# CCR8 Signaling *via* CCL1 Regulates Responses of Intestinal IFN-γ Producing Innate Lymphoid CelIs and Protects From Experimental Colitis

**DOI:** 10.3389/fimmu.2020.609400

**Published:** 2021-02-05

**Authors:** Le Kang, Angelika Schmalzl, Tamara Leupold, Miguel Gonzalez-Acera, Raja Atreya, Markus F. Neurath, Christoph Becker, Stefan Wirtz

**Affiliations:** ^1^ Medizinische Klinik 1, Universitätsklinikum Erlangen, Friedrich-Alexander-Universität Erlangen-Nürnberg, Erlangen, Germany; ^2^ Medical Immunology Campus Erlangen, FAU Erlangen-Nürnberg, Erlangen, Germany

**Keywords:** inflammatory bowel disease, innate immunity, chemokines, cytokines, innate lymphoid cell, CCR8

## Abstract

A diverse spectrum of immune cells populates the intestinal mucosa reflecting the continuous stimulation by luminal antigens. In lesions of patients with inflammatory bowel disease, an aberrant inflammatory process is characterized by a very prominent infiltrate of activated immune cells producing cytokines and chemokines. These mediators perpetuate intestinal inflammation or may contribute to mucosal protection depending on the cellular context. In order to further characterize this complex immune cell network in intestinal inflammation, we investigated the contribution of the chemokine receptor CCR8 to development of colitis using a mouse model of experimental inflammation. We found that CCR8^−/−^ mice compared to wildtype controls developed strong weight loss accompanied by increased histological and endoscopic signs of mucosal damage. Further experiments revealed that this gut protective function of CCR8 seems to be selectively mediated by the chemotactic ligand CCL1, which was particularly produced by intestinal macrophages during colitis. Moreover, we newly identified CCR8 expression on a subgroup of intestinal innate lymphoid cells producing IFN-γ and linked a functional CCL1/CCR8 axis with their abundance in the gut. Our data therefore suggest that this pathway supports tissue-specific ILC functions important for intestinal homeostasis. Modulation of this regulatory circuit may represent a new strategy to treat inflammatory bowel disease in humans.

## Introduction

Inflammatory bowel diseases (IBD) are idiopathic inflammatory disorders of the gastrointestinal tract. Crohn’s disease (CD) and ulcerative colitis (UC) are the two main manifestations of IBD (1). While both diseases are chronic and relapsing inflammatory diseases, they can typically be distinguished by the location of inflammatory lesions in the gastrointestinal tract and by the pattern of histological alterations in the bowel wall. Although the etiology of IBD still remains incompletely understood, it is generally agreed that a complex interplay between genetic, environmental, and immunological factors contributes to disease initiation and progression (2, 3).

Even in the steady state, the intestinal mucosa is populated by a diverse spectrum of immune cells reflecting its continuous stimulation by luminal antigens. However, in lesions of patients with IBD, the aberrant inflammatory process is accompanied by a very prominent infiltrate of immune cells of both the innate and adaptive immune system. While immune cell derived cytokine production is indispensable for mucosal homeostasis and can robustly protect the mucosa from pathogen entry, dysregulated cytokine responses have a crucial role in the pathogenesis of IBD by controlling several aspects of the inflammatory reaction (4). Consistently, the modulation of cytokine responses can have deleterious or therapeutic effects and such therapeutic strategies are of increasing clinical importance (5). Yet, the complex immunological processes that are important for the fine-tuning of cytokine signals during intestinal immune responses are incompletely understood.

Chemokines comprise a group of small cytokines with the capacity to induce directional leukocyte migration. Through binding to their cognate receptors, which belong to the class A rhodopsin-like family of G-protein–coupled receptors, they play fundamental roles in normal physiology as well as in inflammatory and infectious diseases (6). Although nearly any cell type can produce chemokines upon stimulation, immune cells have been demonstrated to be a major source during inflammatory reactions. Besides their manifest capacity to coordinate immune cell migration and positioning, various other regulatory roles during growth, survival, or cytokine production of immune cells have been revealed recently (7). Both experimental work in mouse models and evidence from clinical studies support an important role for several chemokine receptors in the pathogenesis of IBD (8). However, given the multicellular immune cell infiltrate in IBD, the role of several chemokine receptors in the complex chemokine network in the inflamed mucosa remains incompletely defined. The chemokine receptor CCR8 belongs to the family of CC-type chemokine receptors and was initially described as a specific receptor for CCL1/T cell activation-specific gene 3 (TCA3) by inducing calcium flux and migratory responses in CCR8-transfected cell lines (9, 10). Subsequently, mouse CCL8 has been identified to be a second agonist for mouse CCR8, while in humans CCL18, but not CCL8, is a further CCR8-specific chemokine ligand (11, 12). It has been reported that CCR8 is predominantly expressed in T helper type 2 (Th2) cells and that CCL1/CCL8-CCR8 signaling is an important pathway in the pathogenesis of several type 2 related inflammatory diseases including asthma and atopic dermatitis (13, 14). In line with this, recent data also suggest a vital role of CCR8 for the local migration of CD301b^+^ dermal dendritic cells (DC) during cutaneous type 2 immune responses (15). In both humans and mice, CCR8 is also expressed on Foxp3^+^ regulatory T cells and has been significantly implicated in their immunosuppressive functions *in vivo* (16–18). We and others have recently demonstrated strong functionally-relevant expression of CCR8 on mouse and human group 2 innate lymphoid cells (ILC2s) indicating that this receptor is an important regulator of lymphocyte subsets with important functions during early phases of immune responses (19, 20).

Here, we analyzed the role of CCR8 in the context of intestinal inflammation. We found that CCR8 signaling protects mice from acute intestinal damage and that this function is selectively mediated by the ligand CCL1. We newly identified CCR8 expression on a subgroup of intestinal ILCs producing IFN-γ and linked a functional CCL1/CCR8 axis with their abundance in the gut suggesting that this pathway axis supports tissue-specific ILC functions important for intestinal homeostasis.

## Methods

### Animals and Husbandry

*Ccr8* ^−/−^ mice (21) were gratefully provided by F. Tacke (University Hospital Center Aachen, Germany). C57BL/6 mice were initially obtained from Jackson and subsequently bred in house. Sterile drinking water and food were provided *ad libitum*. All animals were kept in individually ventilated cages (IVC), and the health status of the colony was assessed periodically for pathogens in adherence with the guidelines of the Federation of European Laboratory Animal Science Associations. In order to normalize the intestinal microbiota between groups, C57BL/6 mice and *Ccr8* ^−/−^ mice were immediately after weaning co-housed (female) or beddings (male) were exchanged for at least 5 weeks. Metagenomic sequencing of the V3-V4 region of the bacterial 16S gene (22) using DNA isolated from stool samples was used to demonstrate the presence of a similar intestinal microbiome in *Ccr8* ^+/+^ and *Ccr8* ^−/−^ mice (**Supplementary Figure 1**). Eight to 12 weeks old and sex-matched mice were utilized in the experiments. To induce the acute colitis, mice were treated with 2% DSS (MP Biomedicals, Eschwege, Germany) in drinking water for 7 days as described previously (23). Overexpression of chemokines was performed as described previously by us (20). Animal experiments were approved by the local animal ethical committee of the government of Unterfranken, Würzburg, Germany.

### Human Study Subjects

Human gut tissue specimens (N = 30 each) were obtained from CD, UC, and control patients during endoscopy or surgery. Samples were included in the study after obtaining prior written informed consent from each patient and sample collection was previously approved by the ethical committee of the University of Erlangen-Nuremberg (approval number: 249_13).

### Isolation of Gut Single Cells

For the preparation of intraepithelial lymphocytes and leucocyte single cell suspensions from colonic lamina propria, the Lamina Propria Dissociation Kit and a gentleMACS™ Octo Dissociator were used according to the manufacturer’s instructions (Miltenyi Biotec, Germany). Subsequently, cells were further purified using a Percoll gradient. In brief, 80% Percoll (GE Healthcare) was overlayed with 40% Percoll containing leucocytes. After centrifugation at 1,400 rpm for 20 min at room temperature without breaks, interphases were collected and washed in PBS. Cell numbers were determined in a Neubauer-improved counting chamber and single cell suspensions were used for further *ex vivo* phenotyping.

### Flow Cytometry

Gut leucocytes were incubated with anti-CD16/CD32 antibodies (anti-Fc-receptor; eBioscience) prior to specific surface marker and intracellular staining. For ILC identification, specific lineage preclusion was applied. In brief, Fc-receptor-blocked cells were incubated with a custom made biotinylated lineage antibody cocktail (anti-B220, anti-CD3, anti-CD5, anti-GR1, anti-SiglecF, anti-Ter119; Miltenyi Biotec). After washing, cells were passed to regular surface staining including streptavidin-Brilliant-Violet 421 (BV421; BioLegend) or VioBright FITC (Miltenyi) for labeling of biotinylated antibodies. Antibodies were purchased from Miltenyi Biotec if not otherwise indicated. APC (Alexa647), BV510, BV650, FITC, PacificBlue (BV421, VioBlue), PE, PE-Cy7 or PerCP-Cy5.5 (PerCP-efluor710, PerCP-Vio700) conjugated antibodies were used. For surface staining, cells were incubated with different combinations of anti-CD45 (30-F11), anti-CD4 (GK1.5), anti-CD8 (REA601), anti-CD1d (1B1, BioLegend), anti-TCRγδ (GL3, Biolegend), anti-NK1.1 (PK136), anti-NKp46 (29A1.4, eBioscience), anti-CD25 (PC61), anti-CD11b (REA592), anti-CD11c (REA754), anti-SiglecF (REA798), anti-TCRβ (H57-597, BioLegend), anti-KLRG1 (2F1), anti-F4/80 (BM8, eBioscience), anti-Ly6C (BD Biosciences), anti-Ly6G (1A8, Biolegend), anti-Cd49b (DX5, eBioscience), and anti-Thy1.2 (30-H12) antibodies. In order to enable intracellular cytokine staining, cells were stimulated with the 1× Cell Stimulation Cocktail (plus protein transport inhibitors) (eBioscience) for 4 h. For subsequent intracellular staining of transcription factors and/or Interleukins, cells were fixed and permeabilized with the FoxP3 Transcription Factor Staining Buffer according to manufacturer’s instructions (eBioscience). For intracellular staining, antibody combinations of anti-Tbet (eBio4b10, eBioscience), anti-IFNγ (XMG1.2, eBioscience), anti-Gata3 (REA174), anti-IL-22 (Poly5164, BioLegend), anti-RORγt (Q31-378, BD), anti-CCL1 (148113, R&D Systems), and anti-FoxP3 (FJK-16S, eBioscience) were utilized. Because several commercially available anti-mouse CCR8 mAbs were confirmed as unspecific, we applied custom-made fluorochrome-labeled CCL1 proteins to detect CCR8 surface expression (20). Briefly, before labeling with antibodies, cells were incubated with murine CCL1-AF647 (5 nM; Almac) for 1.5 h at 37°C and washed. Samples were analyzed on a LSRFortessa cell analyzer (BD Bioscience) and evaluated with Flowjo 10 (Treestar). For gating strategies of specific immune cell populations, see **Supplementary Figure 2**.

### Histological Methods

Colon samples were fixed in Roti^®^-Histofix 4.5% (Carl-Roth, Germany) and embedded in paraffin. 4-µm slices of each sample were transferred to microscope slides and subsequently stained with hematoxylin and eosin (H&E) to examine immune cell infiltrates and tissue. Microscopy samples were analyzed on a Leica DMI 6000B microscope.

### Gene Expression Analysis

Total RNA was isolated from fresh tissues with the NucleoSpin RNA Plus Kit (Macherey-Nagel, Germany) according to the manufacturer’s instructions. cDNA was synthesized with the Script RT-PCR kit (Jena Bioscience, Germany). Quantitative PCR (qPCR) analyses were performed using predesigned QuantiTect Primer assays (Qiagen) for mouse samples and TaqMan Gene Expression Assays (Thermo Fisher Scientific) for human samples in a CFX96 system (Bio-Rad). To calculate the relative expression of indicated genes, hypoxanthine phosphoribosyltransferase 1 (hprt) was used as the reference gene.

### RNA Sequencing

1 cm of distal colon tissue of DSS-treated mice was snap-frozen and subsequently, total RNA was isolated using the NucleoSpin RNA Plus Kit from Macherey-Nagel on the basis of manufacturer’s instructions. Total RNA was quantified and quality-controlled using a Experion system (Biorad) and sent to BGI Genomics for sequencing on a BGIseq 500 platform. For bioinformatic analysis, paired-end clean reads were mapped to the reference genome (mm10) using HISAT2 (v.2.0.4) software. FeatureCounts (v.1.6.4) was used to count the read numbers mapped to each gene. Differential expression analysis between two conditions with three biological replicates per condition was performed using DESeq2 (v.1.22.1). The resulting p values were adjusted using the Benjamini and Hochberg’s approach for controlling the False Discovery Rate (FDR). Genes with an adjusted p value < 0.05 found by DESeq2 were assigned as differentially expressed. Raw data have been deposited in the NCBI BioProject database (accession code PRJNA679147).

### Enzyme-Linked Immunosorbent Assays

In order to determine specific concentration of CCL1, CCL8, IL-22, and IFN-γ in cell culture supernatants, ELISA Kits from R&D Systems and Ebioscience were applied in line with the manufacturer’s instructions.

### Statistics

Statistical tests were performed using Graph Pad Prism V8 software. If not otherwise indicated, a two-tailed Mann-Whitney U test with 95% confidence interval was performed for comparison of two groups (*P < 0.05; **P < 0.01; ***P < 0.001; NS, not significant).

## Results

### CCR8 Protects From Acute Colitis

**Figure 1:**
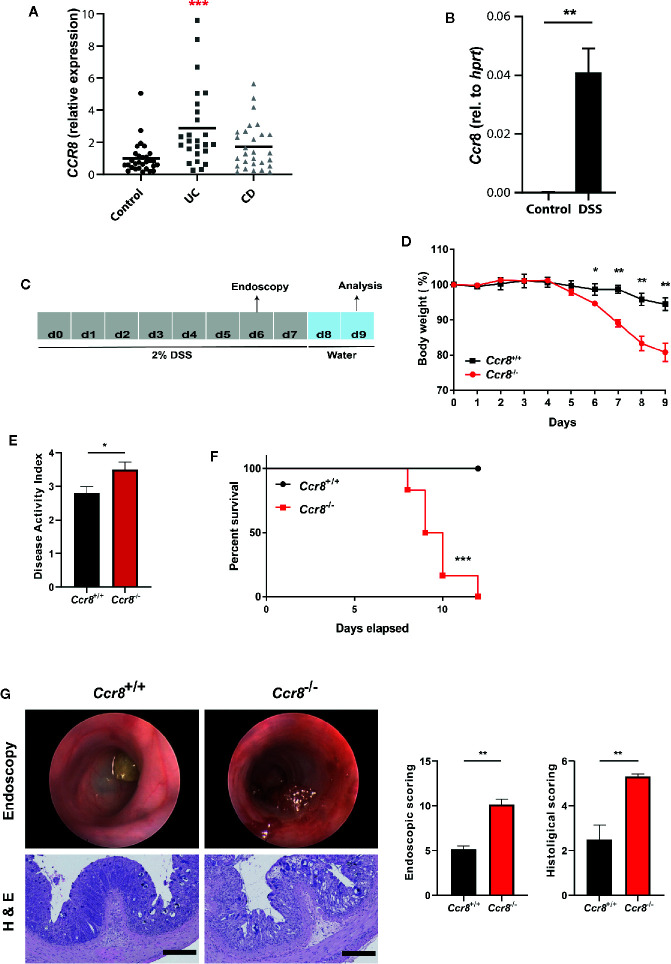
*Ccr8* ^−/−^ mice are highly susceptible to development of DSS colitis. **(A)** CCR8 relative expression in gut biopsies of patients with UC, CD, and controls as determined by qPCR. **(B)** The expression of *Ccr8* was determined in whole colonic tissue lysates of naïve and DSS-treated (day 9) C57BL/6 mice (n = 5/group). **(C)** Schematic overview of the procedure of the DSS treatment protocol. **(D)** Body weight curve throughout the experiment. **(E)** Disease activity index at day 8. **(F)** Survival curves of two groups post 2% DSS treatment. **(G)** Mini-endoscopic images were acquired at day 6 (upper panel). Histological changes were scored in H & E stained paraffin-embedded colonic cross-sections (lower panel). **(A)** Each dot represents one patient. **(D–G)** Graphs show data of one representative experiment out of six independent experiments with four to six mice per group. Statistical analysis was performed using one-way ANOVA **(A)**, Mann-Whitney U test **(B, D, E, G)** or log rank test **(F)**. Data are shown as mean ± S.E.M.; *P ≤ 0.05; **P ≤ 0.01; ***P ≤ 0.001.

In initial expression studies, CCR8 expression was upregulated in the intestinal mucosa of patients with ulcerative colitis and to a lesser extend Crohn’s disease compared to controls as evidenced by qPCR analysis of total RNA of gut tissue specimens (**Figure 1A**). Similarly, mucosal CCR8 transcripts were increased in the context of dextrane sodium sulphate (DSS) induced colitis in mice (**Figure 1B**) indicating that this chemokine receptor might be somehow involved in the development of inflammatory bowel disease. To determine the functional role of CCR8 in the development of intestinal inflammation in mice, microbiota-normalized wildtype control and *Ccr8*-deficient (*Ccr8* ^−/−^) mice were administered with DSS in drinking water for 7 days, a model resembling many features of ulcerative colitis in humans (**Figure 1C**) (23). Following treatment with DSS, *Ccr8* ^−/−^ animals displayed significantly greater body weight loss and increased disease activity indices (DAI) when compared with WT mice (**Figures 1D, E**). Moreover, *Ccr8* ^−/−^ mice exhibited high lethality by day 11, while all WT mice survived in this setting (**Figure 1F**). Consistently, miniendoscopic and histopathological analysis of colonic tissue by hematoxylin and eosin (H&E) staining clearly showed that *Ccr8*-deficiency was accompanied by largely increased inflammatory cell infiltration and by a more severe destruction of the mucosal epithelial layer and the regular colonic crypt structure in response to DSS treatment (**Figure 1G**). Collectively, these data demonstrate that CCR8 expression potently provides protection against DSS-induced intestinal inflammation.

### CCL1 Selectively Protects Mice From DSS Colitis

**Figure 2:**
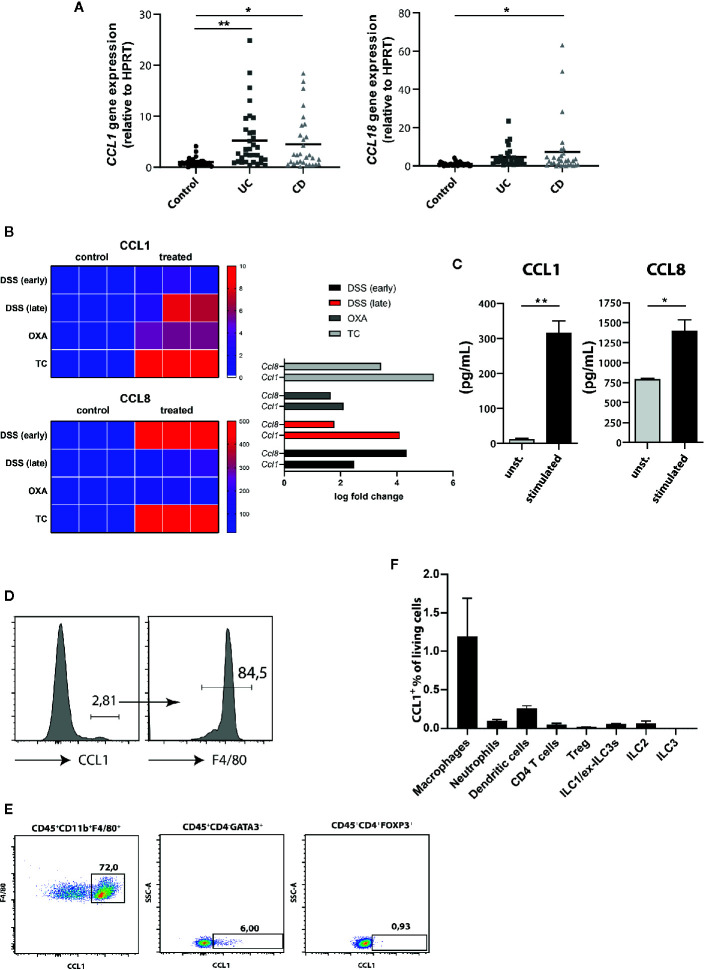
CCR8 ligands are upregulated during intestinal inflammatory conditions. **(A)** CCL1 and CCL18 relative expression in gut biopsies of patients with UC, CD, and controls as determined by qPCR. **(B)** Heat map representation of relative Ccl1 (upper), Ccl8 (lower) expression in dextran sulfate sodium (DSS) (early, d4), DSS (late, d8), Oxazolone (Oxa), Transfer (TC) colitis model (n = 3 per group). **(C)** The concentrations of CCL1 and CCL8 protein in supernatants of colonic C57BL/6 LPMC stimulated with or without Ionomycin and Phorbol 12-myristate 13-acetate (PMA) for 48 h (n = 6/group). **(D–F)** The expression of CCL1 in selected immune cell subsets within LPMC isolated from C57BL/6 mice with DSS colitis (day 9) was analyzed. Representative flow cytometric plots (n = 5/group). Statistical analyses were performed using one-way ANOVA **(A)** or Mann-Whitney U test **(C)**. The results were expressed in mean ± S.E.M. *P ≤ 0.05; **P ≤ 0.01.

Similar to most other chemokine receptors, CCR8 responds to multiple chemokine ligands. We therefore quantified the expression of CCL1 and CCL18, the two known human ligands for CCR8, in intestinal tissue specimens of controls and patients with active IBD by qPCR. Thereby we found significantly increased CCL1 expression in both UC and CD patients, while CCL18 expression was only evident in the mucosa of a smaller subgroup of patients and here most prominently in individuals with CD (**Figure 2A**). Likewise, data from RNAseq experiments confirmed that transcripts of CCL1 and CCL8, the murine homolog of CCL18, were upregulated across several mouse models of intestinal inflammation relative to normal colon samples (**Figure 2B**). Moreover, increased CCL1 and CCL8 protein concentrations were present in supernatants of stimulated lamina propria mononuclear cells (LPMCs) isolated from mice with DSS colitis (**Figure 2C**). Although lower compared to unspecific stimulation with PMA/Ionomycin, stimulation with the TLR9 ligand CpG1668 or a combination of LPS and ATP increased CCL1 in LPMC supernatants (**Supplementary Figure 3**). Notably, flow cytometry studies identified intestinal macrophages as important producer of CCL1 (**Figure 2D**) compared to ILC2s (20) or regulatory T cells (16) or other important immune cells within LPMC of mice with DSS colitis (**Figures 2E, F**) confirming previous results that found CCL1 protein in exosomes of gut-derived M2 macrophages (24). Similarly, CCL8 expression in the course of DSS colitis was localized to intestinal macrophages in earlier studies (25).

**Figure 3:**
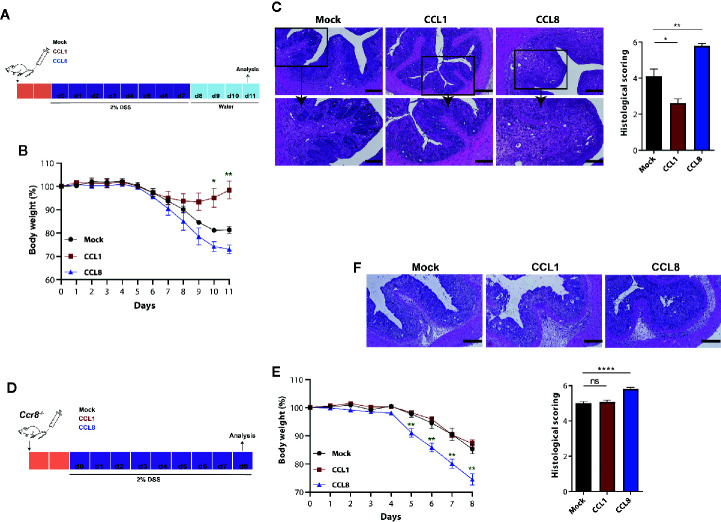
CCL1 but not CCL8 protects from DSS colitis. C57BL/6 (A-C) or *Ccr8* ^−/−^ (D-F) mice were intravenously treated with 5 μg of expression vectors (CCL1, CCL8, and Mock). Two days later, the mice were treated with 2% of DSS in drinking water for 7 days. **(A, D)** Schematic overview of the procedure. **(B, E)** Relative body weight. **(C, F)** Representative H&E stainings of colonic cross sections and histological scoring of mucosal damage. Scale bar 500 µm and 200 µm. All graphs show data of one representative experiment out of three to four independent experiments with four to six mice per group. Statistical analyses were performed using one-way ANOVA. Data is shown as mean ± S.E.M.: *P ≤ 0.05; **P ≤ 0.01; ****P ≤ 0.0001, ns, not significant.

To ascertain the individual capacity of both CCR8 ligands to regulate mucosal damage during acute colitis, we studied in the next series of experiments the development of DSS colitis in the absence or presence of systemic CCL1 or CCL8 overexpression. To this end, C57BL/6 wildtype mice were intravenously-treated with minicircle-based expression vectors as described previously (20) 2 days prior oral DSS treatment (**Figure 3A**). Compared to the control group (Mock), in which DSS treatment led to a rapid body weight loss from days 5 to 11, CCL1 overexpression markedly blocked wasting disease (**Figure 3B**) and mice developed only mild histological signs of inflammation. By contrast, CCL8 overexpressing mice suffered from severe colitis characterized by profound loss of crypt structure, edema formation and inflammatory cell infiltrations (**Figure 3C**). We furthermore increased in a similar manner the systemic abundance of CCL1 and CCL8 in *Ccr8* ^−/−^ mice before treatment with DSS (**Figure 3D**). In this setting, CCL8 treatment resulted in increased colitis even in the absence of CCR8 (**Figures 3E, F**) supporting the concept that this chemokine seemingly at least under these particular experimental settings employs other CCR8 independent signal transduction pathways to amplify intestinal inflammation *in vivo*. Conversely, CCL1 treated mice displayed similar susceptibility to mucosal damage as control-treated *Ccr8* ^−/−^ mice consistent with a gut protective functional role of CCL1/CCR8 signaling during experimental colitis.

### Decreased Expression of Type II IFN Signature Genes in *Ccr8*-Deficient Mice With Colitis

**Figure 4:**
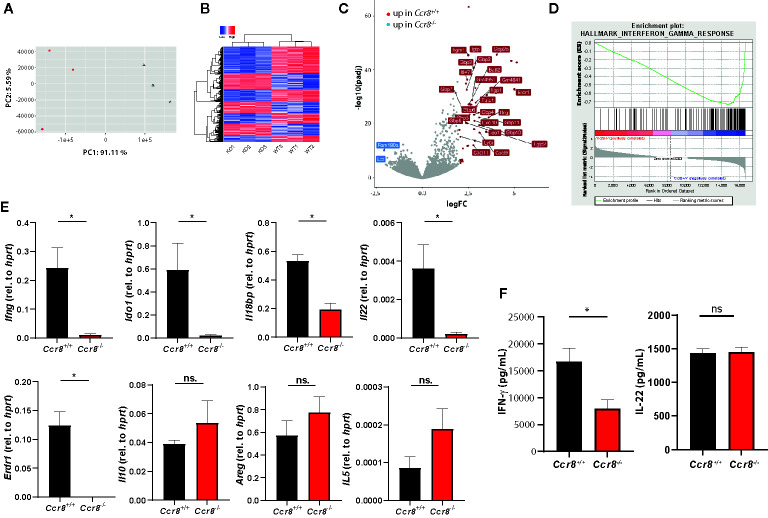
Decreased expression of type II IFN signature genes in Ccr8-deficient mice with colitis. Ccr8^+/+^ and Ccr8^−/−^ mice were subjected to the oral DSS treatment for 7 days. Total RNA of distal colonic specimens (day 9) was isolated and used for bulk RNAseq analysis. **(A)** Principal component analysis (PCA) of total variation in differentially expressed genes. **(B)** Hierarchical cluster analysis of differentially expressed genes among Ccr8^+/+^ and Ccr8^−/−^ mice. **(C)** Volcano plot representation of gene expression changes. **(D)** Gene set enrichment analysis (GSEA) showing enrichment in IFN-γ regulated genes in *Ccr8* ^+/+^ mice compared to *Ccr8* ^−/−^ mice. N = 3 mice/group. **(E)** The transcripts of selected genes in colonic tissue lysates were determined by specific qPCR. The data are represent one of three independent experiment (n = 3–5/group). **(F)** The concentration of IFN-γ and IL-22 in cell culture supernatants of LPMC stimulated for 48 h with PMA/Ionomycin was determined by ELISA. Pooled data from two independent experiments (n = 6/group). Statistical analysis was performed using Mann-Whitney U test. Results represent means ± S.E.M.: *P ≤ 0.05, ns, not significant.

We next used RNAseq-based gene expression profiling to investigate the impact of Ccr8 inactivation on the global colonic transcriptome in mice with DSS colitis. Principal component analysis (PCA) and unsupervised hierarchical clustering of gene expression counts showed clustering of CCR8-deficient mice away from the wildtype tissue samples indicating genotype-associated gene expression patterns that might relate to the differences in the extent of intestinal inflammation (**Figures 4A, B**). Moreover, comparative analysis of the response of control and *Ccr8^−/−^* mice to DSS-treatment identified a number of genes specifically upregulated in wildtype mice, while at the same time only a few genes were significantly upregulated in intestinal tissues of *Ccr8* ^−/−^ mice (**Figure 4C**). Interestingly, further analysis including gene set enrichment analysis revealed in the list of genes downregulated in the absence of CCR8, the enrichment of IFN-γ and several other genes known to be induced by interferon-signaling. These included a number of guanylate binding proteins (Gbps), chemokines (CXCL9, CXCL10, CXCL11), basic leucine zipper transcription factor (Batf) 2, erythroid differentiation regulator (Erdr) 1 and Indoleamine 2,3-Dioxygenase (IDO) 1 (**Figures 4C, D**). The differential regulation of some of these genes was confirmed *via* qPCR, while other genes previously implicated in CCR8 signaling in Treg or ILC2s such as IL-10, amphiregulin (AREG) and IL-5 were not differentially expressed in both genotypes (**Figure 4E**). Consistent with these gene expression data, we found decreased concentrations of IFN-γ protein in supernatants of stimulated lamina propria mononuclear cells (LPMC) of *Ccr8* ^−/−^ mice compared to controls, while IL-22 concentrations were similar between both genotypes (**Figure 4F**).

### CCL1/CCR8 Signaling Supports Mucosal Innate Lymphoid Cells IFN-γ Production

**Figure 5:**
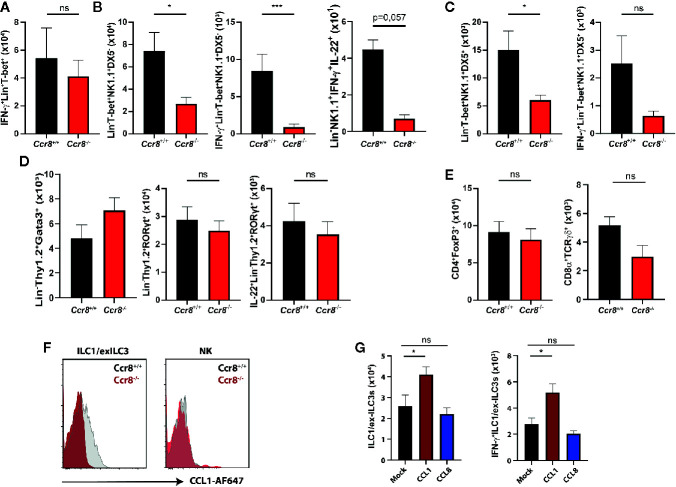
CCL1/CCR8 signaling affects intestinal IFN-γ producing ILC1/ex-ILC3s. **(A–E)** LPMC cells were isolated from *Ccr8* ^+/+^ and *Ccr8* ^−/−^ mice subjected to DSS-treatment (day 9), stimulated for 4 h with PMA/Ionomycin in the presence of a protein transport inhibitor and analyzed by flow cytometry. **(F)** Representative flow cytometric plots of CCR8 expression on ILC1s and NK cells using fluorophore-coupled CCL1 (CCL1-AF647) in *Ccr8* ^+/+^ and *Ccr8* ^−/−^ mice. **(G)** LPMC cells were isolated from C57BL/6 mice overexpressing CCL1 or CCL8 subjected to DSS-treatment (day 9), stimulated for 4 h with PMA/Ionomycin in the presence of a protein transport inhibitor and analyzed by flow cytometry. Pooled data of two independent experiments with at least four mice in each group. Statistical analysis was performed using the Mann-Whitney U test or one-way ANOVA and the results were expressed in mean ± S.E.M.: *P ≤ 0.05; ***P ≤ 0.001, ns, not significant.

Excessive IFN-γ production has been implicated in mucosal damage and the pathogenesis of IBD in mice and humans (26, 27). However, it has also been shown that IFN-γ signaling in the gut promotes the expression of important regulators of intestinal immune homeostasis (28–31) suggesting that a tightly controlled IFN-γ production by immune cells is important to maintain the delicate balance between immune tolerance to commensal microbes and effective immune responses to pathogens or pathobionts. We therefore next compared the frequencies of IFN-γ-producing lymphocytes in LPMC of *Ccr8* ^+/+^ and *Ccr8* ^−/−^ mice with DSS colitis using flow cytometry. While there were no clear differences in Lin^+^Tbet^+^ cells, which include Th1 cells, CD8^+^ T cells and NKT cells (**Figure 5A**), we found in colonic tissue decreased numbers of IFN-γ producing innate lymphocytes in the absence of CCR8. Here, a population of Lin^-^Tbet^+^NK1.1^+^DX5^-^ cells that comprise ILC1/exILC3s was significantly decreased in *Ccr8* ^−/−^ mice. Noteworthy, some IFN-γ expressing ILCs co-expressed IL-22, but their numbers were low in comparison. (**Figure 5B**). The numbers of Lin^-^Tbet^+^NK1.1^+^DX5^+^ natural killer (NK) cells producing IFN-γ tended to be reduced in these mice (**Figure 5C**), while the frequencies of other ILCs (Lin^-^Thy1^+^Gata3^+^ ILC2s, Lin^-^Thy1^+^Rorgt^+^ ILC3s) (**Figure 5D**) as well as CD4^+^Foxp3^+^ regulatory T cells and TCRγδ^+^ T cells (**Figure 5E**) were similar in both genotypes. In line with this finding, we were able to detect CCR8 on the surface of gut ILC1-like cells by flow cytometric analysis using fluorescence-labeled CCL1 proteins and cells from *Ccr8* ^−/−^ mice as controls. However, although CCR8 has been previously found to be expressed in human NK cells, intestinal NK cells stained negative for this receptor (**Figure 5F**). In further experiments, we also analyzed, whether increased *in vivo* concentrations of CCL1 or CCL8 affected the mucosal frequencies of IFN-γ producing ILCs in the lamina propria of mice with DSS colitis. Notably, in this gain of function approach CCL1 but not CCL8 overexpression increased the frequencies of gut ILC1/exILC3 populations indicating that CCL1/CCR8 signaling supports mucosal innate lymphoid cells IFN-γ production (**Figure 5G**).

**Figure 6:**
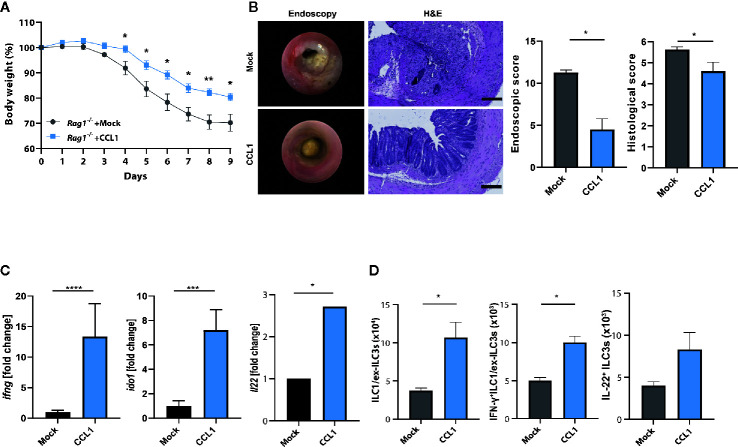
CCL1/CCR8 axis protects from colitis in the absence of adaptive immune cells. *Rag1* ^−/−^ mice were intravenously treated with 5 μg of CCL1 or control expression vectors. Two days later, the mice were exposed to 2% of DSS in drinking water for 7 days. **(A)** Relative body weight. **(B)** Representative H&E stainings of colonic cross sections and endoscopic pictures. Scale bar 100 µm. Histological and endoscopic scoring of mucosal damage. **(C)** Transcripts of IFN-γ and amphiregulin in colonic tissue lysates were determined by specific qPCR. **(D)** LPMC cells were isolated, stimulated for 4 h with PMA/Ionomycin in the presence of a protein transport inhibitor and analyzed by flow cytometry. The graphs show data of one representative experiment out of two independent experiments with four to six mice per group. Statistical analyses were performed using the Mann-Whitney U test. Data represent means ± S.E.M. n≥4 per group: *P ≤ 0.05; **P ≤ 0.01; ***P ≤ 0.001.

To establish further evidence that the protective role of CCL1/CCR8 signaling during acute colitis is primarily driven by innate immune system mediated mechanisms, we overexpressed CCL1 in lymphopenic Rag1^−/−^ mice, which lack T and B cells, but harbor a functional ILC compartment. Interestingly, mice overexpressing CCL1 showed significantly less colitis-dependent weight loss (**Figure 6A**) consistent with reduced endoscopic and histological signs of disease (**Figure 6B**). Moreover, increased expression of CCL1, was related to increased mucosal IFN-γ transcription, its target gene Ido1 and also IL-22 (**Figure 6C**). In addition, increased frequencies of ILC subsets were present in LPMC of these mice (**Figure 6D**).

Collectively, these findings infer that activation of CCR8 by CCL1 during intestinal inflammation alters the colonic microenvironment. This pathway protects from the development of innate colitis and is linked to transcriptional programs driven by IFN-γ produced by innate lymphocytes.

## Discussion

Healthy tissue environments are able to provide fine-tuned immune responses by proper coordination of the migration, local positioning and activation of leukocytes. Given the multicellular immune cell infiltrate in IBD, chemokines and their cognate receptors are most likely of foreseeable importance for the pathophysiology of these severe diseases. Indeed, data from mouse models and studies with tissue from patients with IBD suggests important roles for several chemokine receptors in the development of intestinal inflammation (8). In this study here, we explored the functional role of the CC chemokine receptor CCR8 and its activation by the two known ligands CCL1 and CCL8 in DSS colitis, a widely used mouse model of IBD resembling important features of UC. Different from the closely related molecule CCR4, which has been previously shown to drive pathogenic inflammatory reactions in mouse colitis models (32), *Ccr8* ^−/−^ mice developed highly increased mucosal damage compared to control mice. Interestingly, further experiments clearly suggested that these CCR8-mediated tissue protective effects are exclusively mediated by the ligand CCL1, while CCL8-treatment of wildtype mice rather exacerbated colitis. In line with the latter observation, Asano et al. demonstrated that an antibody-mediated strategy to neutralize CCL8 protected mice from DSS colitis. In this study, high CCL8 expression was located to lamina propria resident CD169^+^ macrophages and required for chemo-attraction of inflammatory monocytes (25). It remains unclear at present, how such different roles of CCL1 and CCL8 could be explained. Many chemokine receptors can be stimulated by more than one chemokine ligand, and there is emerging evidence that signaling of individual ligands can result in differential functional responses. Such functional selectivity/biased agonism has been described for several other important chemokine receptor ligands including CCR4 (33, 34). In the case of CCR8, functional selectivity has not been studied in depth, yet recent studies by us and others indicate that CCL1 and CCL8 differentially regulate biological functions of ILC2s (19, 20). However, CCL8-injected *Ccr8* ^−/−^ mice still displayed higher mucosal damage than control mice indicating that these proinflammatory effects are mediated by signaling to one or more further chemokine receptors. Indeed, most chemokine receptors bind to more than one cellular receptor subtype to mediate their biological functions and are often highly promiscuous with regard to ligand specificity (35). Whether CCL8 employs further receptors in addition to CCR8 remains to be determined in future studies.

Treatment of mice with DSS leads to a compromised barrier integrity and subsequently exposes mucosal immune cells to luminal antigens resulting in a rapid and profound inflammatory immune response. The appearance of strong colitis in lymphopenic mice indicates the importance of effector mechanism not depending on the presence of adaptive immune cells (36). Nonetheless, T cells have been demonstrated to accumulate in the inflamed mucosa over time in DSS colitis, where they appear to have a pathogenic role or in the case of Treg help to suppress colitis (37, 38). Because CCR8 is expressed on Foxp3^+^ Treg and has been implicated in their suppressive capacity in autoimmunity and cancer, it was tempting to speculate that dysregulated Treg functions contributed to exacerbated DSS colitis in *Ccr8* ^−/−^ mice (16). However, we observed in the acute DSS model no changes in intestinal Foxp3^+^ Treg frequencies or the production of the immunosuppressive Treg effector cytokine IL-10 in Ccr8^−/−^ mice. Moreover, CCL1-treatment of Rag1^−/−^ mice ameliorated intestinal inflammation indicating that CCR8-dependent innate immune responses are critical mediators of the tissue protective capacity of the CCL1/CCR8 axis. Possibly, CCR8 expressing Treg are more prominent immunoregulatory factors in T cell dependent models of colitis such as chronic DSS colitis or T cell transfer colitis. Indeed we observed upregulation of CCL1 expression in the latter model and oxazolone colitis suggesting that this chemokine plays broad immunoregulatory roles in various settings of experimental colitis and potentially IBD in humans. In the innate immune cell compartment, the numbers of innate lymphocytes were reduced in the lamina propria of *Ccr8* ^−/−^ mice with DSS colitis compared to controls. This only recently characterized cell population modulates immune responses and tissue homeostasis at multiple levels and is particularly enriched in barrier surfaces such as the gut (39, 40). In previous studies we found that ILC2s, the innate counterpart of T helper type 2 cells, display compared to all other immune cells we studied the highest levels of surface CCR8 expression (20). Even though Monticelli et al. reported protective roles of the ILC2-derived growth factor amphiregulin during intestinal damage repair (41), we observed no significant changes in the intestinal ILC2 compartment in *Ccr8* ^−/−^ mice. In addition, our in depth analysis of ILC2-deficient mice revealed no major tissue protective role of these cells in the DSS model. Conversely, we identified that *Ccr8*-deficiency was clearly associated with reduced intestinal frequencies of ILCs expressing the transcription factor T-bet and upon stimulation the cytokine IFN-γ. Accordingly, these cells, which are most likely bona fide ILC1s or so-called ex-ILC3s that lost expression of ROR-γt, express CCR8 protein and are increased by treatment of mice with CCL1. Interestingly, our data indicate that CCL1 not only regulates innate type 2 responses, which are broadly implicated in tissue repair processes, but also cells related to the innate type 1 response. During type 2 polarized immune responses e.g. induced by parasitic infections or atopic diseases, CCL1 has been shown to be primarily produced by mast cells and ILC2s (20, 42) most likely by stimulation *via* alarmins, such as IL-33. In the context of DSS colitis our data support previous findings that macrophages are dominant producers of CCL1 (24, 43). This indicates that the spatiotemporal context of CCL1 expression as well as local factors driving its release by distinct subsets of immune cells are important for the immunoregulatory role of this chemokine. Further studies are therefore necessary to ascertain whether the increased expression of the Ccr8 receptor and its ligands CCL1 and CCL18 in human IBD is contributing to type 2 immunity mediated tissue repair or supports innate type 1 immunity, which has been implicated in CD pathophysiology (44).

Although their functions in the gut are complex and there is a high degree of plasticity between individual subpopulations, there is a substantial body of literature highlighting the critical role of ILC1s and ILC3s in the regulation of the critical balance between maintenance and loss of intestinal homeostasis (45). Indeed, mainly by secreting cytokines but also through cell contact mediated mechanisms they control barrier integrity, provide containment of commensals to the gut lumen and are vital effector cells in immunity against proteobacterial pathogens such as *Citrobacter rodentium* or *Salmonella Typhimurium* (46). Notably, we observed a selective reduction of Tbet^+^ ILCs in colitic *Ccr8* ^−/−^ mice, while the numbers of Rorγt^+^ ILC3s were seemingly not affected. Given that rather NKp46^+^ ILC1s than ILC3s have been shown to be necessary for the control of DSS colitis (47), this indicates that reduced frequencies of ILC1s are causatively involved in the exacerbated disease phenotype in *Ccr8* ^−/−^ mice. In line with their gut protective functions, mice lacking these ILCs have alterations in cecal homeostasis and suffer from bacterial infection in the absence of adaptive lymphocytes (48, 49). Whether CCL1/CCR8 signaling supports pathogenic functions of ILCs that have been reported in e.g. in *anti*-*CD40*-induced innate colitis (44, 48) remains to be determined. In addition, conditional *Ccr8*-deficient mice will help to further characterize the role of CCL1/CCR8 signaling for regulation of ILCs *in vivo* and to identify its cell-type specific functions in the complex immune cell network regulation of intestinal inflammation.

The secretion of the cytokine IFN-γ is a characteristic feature of Tbet^+^ ILC1, while Tbet^+^ exILC3s have been shown to produce much less of the cytokine IL-22 after conversion (50). IL-22 is a pleiotropic cytokine with broad immunoregulatory functions during intestinal infection and inflammation (51). Given that the frequencies of IL-22^+^ ILC3s are not altered in *Ccr8* ^−/−^ mice with DSS colitis and IL-22^+^IFN-γ^+^ exILC3s comprised only a minor fraction of Tbet^+^ ILCs, our results rather indicate that a lack of the anti-inflammatory function of this cytokine did not largely contributes to severe disease development. However, reduced frequencies of ILCs In the inflamed colon of *Ccr8* ^−/−^ mice were linked to lower presence of IFN-γ protein and, as revealed by analysis of the global colonic transcriptome by bulk RNAseq, to the reduced expression of a number of important target genes of IFN-γ signaling. The pleiotropic roles of this potent cytokine in the gut are complex and still incompletely understood. This is exemplified by the fact that depending on the mouse model and on the studied cell population, IFN-γ has been reported as proinflammatory, redundant or even anti-inflammatory contributor to intestinal inflammation (29, 52). Thereby, even in the DSS model the role of IFN-γ is controversial most likely reflecting different housing conditions and/or compositional differences in the intestinal microbiome. For example IFN-γ deficient mice were protected in the studies by Nava et al. and Ito et al. (26, 53), while in other studies this strain developed significantly higher weight loss and mucosal damage than wildtype controls (28, 30). Our study with mice supports the notion that ILC-derived IFN-γ has important tissue protective functions in acute DSS colitis. Noteworthy, transcripts of IL-18 binding protein and IDO1 were highly downregulated in *Ccr8* ^−/−^ mice with colitis. These factors are known to be strongly induced by IFN-γ signaling in intestinal epithelial and other cells and have previously shown to operate as fine tuners of inflammatory responses in the gut and are thus potential mediators of the gut protective functions of CCR8 signaling during intestinal tissue repair (54–56). However, antibody-mediated IL-18 neutralization did not rescue *Ccr8* ^−/−^ from severe intestinal pathology (**Supplementary Figure 3B**) indicating that excessive IL-18 signaling is not a main driver of colitis in this strain.

Although mature intestinal ILCs are mainly tissue resident, their local migration and accumulation upon tissue injury is believed to highly impact their capacity to support repair processes (57). NCR^+^ILCs and ILC2s are dispersed in the lamina propria or in the case of intraepithelial ILC1s positioned within the epithelial layer (58). Whether CCL1 controls anatomical compartmentalization of CCR8^+^ intestinal ILCs in the steady state and intestinal inflammation remains an important open question. The availability of sensitive antibodies for specific CCR8 staining or reporter mouse strains will help to elucidate, whether CCL1 secretion by macrophages represents a migratory signal to guide ILCs to e.g. inflammatory foci. Similarly, CCR8 expression on CD301b^+^ DCs was shown to be essential for migration of these cells from the subcapsular sinus to the parenchyma within lymph nodes (15). This effect was dependent on the ligand CCL8, which also promoted ILC2 trafficking and motility within inflamed lungs. Conversely, autocrine CCL1 production was shown to enhance proliferation of CCR8^+^ Treg and ILC2s indicating that CCL1 secretion could also promote ILC1/exILC3 activation or proliferation.

In summary, we identified CCL1 signaling *via* the cognate chemokine receptor CCR8 as a critical regulatory pathway promoting mucosal homeostasis after intestinal epithelial damage. We described intestinal ILC subsets as novel innate immune cell population regulated by CCL1/CCR8 and implicate their capacity to produce IFN-γ in the tissue protective role of this pathway. Modulation of this regulatory circuit may therefore represent, in combination with other therapeutic strategies, a new method to treat inflammatory bowel disease in humans.

## Data Availability Statement

The data sets presented in this study can be found in online repositories. The names of the repository and accession number can be found here: NCBI SRA; accession number: PRJNA679147.

## Ethics Statement

The studies involving human participants were reviewed and approved by Ethik-Kommission der Friedrich-Alexander Universität Erlangen-Nürnberg. The patients/participants provided their written informed consent to participate in this study. The animal study was reviewed and approved by Regierung Unterfranken.

## Author Contributions

LK, AS, and TL performed experiments and analyzed data. MG-A analyzed data. RA collected human samples. CB and MN discussed the data and critically reviewed the manuscript. SW conceptualized the project and wrote the paper. All authors contributed to the article and approved the submitted version.

## Funding

This work was supported by funds from the German Research Foundation (DFG) (TRR241 A03; TRR241 INF; FOR2886 TP1). The present work was performed in (partial) fulfillment of the requirements for obtaining the degree Dr. med. for Le Kang. Cell sorting was supported by the FACS Core Unit at the Nikolaus-Fiebiger-Center, Erlangen. The authors declare no competing financial interests.

## Conflict of Interest

The authors declare that the research was conducted in the absence of any commercial or financial relationships that could be construed as a potential conflict of interest.

## Supplementary Material

Supplementary Figure 1 Fecal pellets of wildtype and Ccr8^−/−^ mice cohoused for 4 weeks were collected. Genomic DNA was isolated and used for 16S-based metagenomic sequencing. **(A)** PcoA plot showing beta-diversity (bray curtis dissimilarity). **(B)** Alpha diversity (Chao1 index). N = 4–6 mice/group.

Supplementary Figure 2 Gating strategies for flow cytometric characterization of different lymphocyte populations within the mouse intestinal lamina propria.

Supplementary Figure 3
**(A)** The concentrations of CCL1 protein in supernatants of colonic *Ccr8* ^+^ LPMCs stimulated with LPS (1 µg/ml), CpG1668 (1 µm), polyIC (1 µg/ml), Flagellin (0,1 µg/ml), Lipoteichoic acid (LTA; 2 µg/ml), ATP (2,5 mM), IFN-γ (10 ng/ml) IL-1-β (10 ng/ml) alone or in combination or with Ionomycin and Phorbol 12-myristate 13-acetate (PMA) for 48 h (N = 4–6/group). **(B)** *Ccr8* ^−/−^ mice were exposed to 2% of DSS in drinking water for 7 days. At days 4 and 7, they were i.p. treated with 200 mg of anti-IL-18 (clone YIGIF74-1G7) mABs or control antibodies. (N = 4/group).

## References

[1] Xavier RJ Podolsky DK Unravelling the pathogenesis of inflammatory bowel disease. Nature (2007) 448:427–34. 10.1038/nature06005 17653185

[2] Wirtz S Neurath MF Mouse models of inflammatory bowel disease. Advanced Drug Deliv Rev (2007) 59:1073–83. 10.1016/j.addr.2007.07.003 17825455

[3] Maloy KJ Powrie F Intestinal homeostasis and its breakdown in inflammatory bowel disease. Nature (2011) 474:298–306. 10.1038/nature10208 21677746

[4] Bamias G Cominelli F Cytokines and intestinal inflammation. Curr Opin Gastroenterol (2016) 32:437–42. 10.1097/MOG.0000000000000315 27673380

[5] Bevivino G Monteleone G Advances in understanding the role of cytokines in inflammatory bowel disease. Expert Rev Gastroenterol Hepatol (2018) 12:907–15. 10.1080/17474124.2018.1503053 30024302

[6] Bachmann MF Kopf M Marsland BJ Chemokines: more than just road signs. Nat Rev Immunol (2006) 6:159–64. 10.1038/nri1776 16491140

[7] Griffith JW Sokol CL Luster AD Chemokines and chemokine receptors: positioning cells for host defense and immunity. Annu Rev Immunol (2014) 32:659–702. 10.1146/annurev-immunol-032713-120145 24655300

[8] Singh UP Singh NP Murphy EA Price RL Fayad R Nagarkatti M Chemokine and cytokine levels in inflammatory bowel disease patients. Cytokine (2016) 77:44–9. 10.1016/j.cyto.2015.10.008 PMC4666758 26520877

[9] Tiffany HL Lautens LL Gao JL Pease J Locati M Combadiere C Identification of CCR8: a human monocyte and thymus receptor for the CC chemokine I-309. J Exp Med (1997) 186:165–70. 10.1084/jem.186.1.165 PMC2198957 9207005

[10] Roos RS Loetscher M Legler DF Clark-Lewis I Baggiolini M Moser B Identification of CCR8, the receptor for the human CC chemokine I-309. J Biol Chem (1997) 272:17251–4. 10.1074/jbc.272.28.17251 9211859

[11] Islam SA Chang DS Colvin RA Byrne MH McCully ML Moser B Mouse CCL8, a CCR8 agonist, promotes atopic dermatitis by recruiting IL-5+ T(H)2 cells. Nat Immunol (2011) 12:167–77. 10.1038/ni.1984 PMC3863381 21217759

[12] Islam SA Ling MF Leung J Shreffler WG Luster AD Identification of human CCR8 as a CCL18 receptor. J Exp Med (2013) 210:1889–98. 10.1084/jem.20130240 PMC3782048 23999500

[13] Mikhak Z Fukui M Farsidjani A Medoff BD Tager AM Luster AD Contribution of CCR4 and CCR8 to antigen-specific T(H)2 cell trafficking in allergic pulmonary inflammation. J Allergy Clin Immunol (2009) 123:67–73.e3. 10.1016/j.jaci.2008.09.049 19062085 PMC2782398

[14] Panina-Bordignon P Papi A Mariani M Di Lucia P Casoni G Bellettato C The C-C chemokine receptors CCR4 and CCR8 identify airway T cells of allergen-challenged atopic asthmatics. J Clin Invest (2001) 107:1357–64. 10.1172/JCI12655 PMC209325 11390417

[15] Sokol CL Camire RB Jones MC Luster AD The Chemokine Receptor CCR8 Promotes the Migration of Dendritic Cells into the Lymph Node Parenchyma to Initiate the Allergic Immune Response. Immunity (2018) 49:449–63.e6. 10.1016/j.immuni.2018.07.012 30170811 PMC6192021

[16] Barsheshet Y Wildbaum G Levy E Vitenshtein A Akinseye C Griggs J CCR8(+)FOXp3(+) Treg cells as master drivers of immune regulation. Proc Natl Acad Sci USA (2017) 114:6086–91. 10.1073/pnas.1621280114 PMC5468670 28533380

[17] Soler D Chapman TR Poisson LR Wang L Cote-Sierra J Ryan M CCR8 expression identifies CD4 memory T cells enriched for FOXP3+ regulatory and Th2 effector lymphocytes. J Immunol (2006) 177:6940–51. 10.4049/jimmunol.177.10.6940 17082609

[18] Villarreal DO L’Huillier A Armington S Mottershead C Filippova EV Coder BD Targeting CCR8 Induces Protective Antitumor Immunity and Enhances Vaccine-Induced Responses in Colon Cancer. Cancer Res (2018) 78:5340–8. 10.1158/0008-5472.CAN-18-1119 30026324

[19] Puttur F Denney L Gregory LG Vuononvirta J Oliver R Entwistle LJ Pulmonary environmental cues drive group 2 innate lymphoid cell dynamics in mice and humans. Sci Immunol (2019) 4. 10.1126/sciimmunol.aav7638 PMC6744282 31175176

[20] Knipfer L Schulz-Kuhnt A Kindermann M Greif V Symowski C Voehringer D A CCL1/CCR8-dependent feed-forward mechanism drives ILC2 functions in type 2-mediated inflammation. J Exp Med (2019) 216:2763–77. 10.1084/jem.20182111 PMC6888976 31537642

[21] Chensue SW Lukacs NW Yang TY Shang X Frait KA Kunkel SL Aberrant in vivo T helper type 2 cell response and impaired eosinophil recruitment in CC chemokine receptor 8 knockout mice. J Exp Med (2001) 193:573–84. 10.1084/jem.193.5.573 PMC2193397 11238588

[22] Pickert G Wirtz S Matzner J Ashfaq-Khan M Heck R Rosigkeit S Wheat Consumption Aggravates Colitis in Mice via Amylase Trypsin Inhibitor-mediated Dysbiosis. Gastroenterology (2020) 159:257–72.e17. 10.1053/j.gastro.2020.03.064 32251667

[23] Wirtz S Popp V Kindermann M Gerlach K Weigmann B Fichtner-Feigl S Chemically induced mouse models of acute and chronic intestinal inflammation. Nat Protoc (2017) 12:1295–309. 10.1038/nprot.2017.044 28569761

[24] Yang R Liao Y Wang L He P Hu Y Yuan D Exosomes Derived From M2b Macrophages Attenuate DSS-Induced Colitis. Front Immunol (2019) 10:2346. 10.3389/fimmu.2019.02346 31749791 PMC6843072

[25] Asano K Takahashi N Ushiki M Monya M Aihara F Kuboki E Intestinal CD169(+) macrophages initiate mucosal inflammation by secreting CCL8 that recruits inflammatory monocytes. Nat Commun (2015) 6:7802. 10.1038/ncomms8802 26193821 PMC4518321

[26] Ito R Shin-Ya M Kishida T Urano A Takada R Sakagami J Interferon-gamma is causatively involved in experimental inflammatory bowel disease in mice. Clin Exp Immunol (2006) 146:330–8. 10.1111/j.1365-2249.2006.03214.x PMC1942055 17034586

[27] Rovedatti L Kudo T Biancheri P Sarra M Knowles CH Rampton DS Differential regulation of interleukin 17 and interferon gamma production in inflammatory bowel disease. Gut (2009) 58:1629–36. 10.1136/gut.2009.182170 19740775

[28] Muzaki ARBM Tetlak P Sheng J Loh SC Setiagani YA Poidinger M Intestinal CD103(+)CD11b(-) dendritic cells restrain colitis via IFN-gamma-induced anti-inflammatory response in epithelial cells. Mucosal Immunol (2016) 9:336–51. 10.1038/mi.2015.64 PMC4801902 26174764

[29] Thelemann C Eren RO Coutaz M Brasseit J Bouzourene H Rosa M Interferon-gamma induces expression of MHC class II on intestinal epithelial cells and protects mice from colitis. PloS One (2014) 9:e86844. 10.1371/journal.pone.0086844 24489792 PMC3904943

[30] Siegmund B Sennello JA Lehr HA Senaldi G Dinarello CA Fantuzzi G Frontline: interferon regulatory factor-1 as a protective gene in intestinal inflammation: role of TCR gamma delta T cells and interleukin-18-binding protein. Eur J Immunol (2004) 34:2356–64. 10.1002/eji.200425124 15307168

[31] Kuhl AA Pawlowski NN Grollich K Loddenkemper C Zeitz M Hoffmann JC Aggravation of intestinal inflammation by depletion/deficiency of gammadelta T cells in different types of IBD animal models. J Leukocyte Biol (2007) 81:168–75. 10.1189/jlb.1105696 17041003

[32] Heiseke AF Faul AC Lehr HA Forster I Schmid RM Krug AB CCL17 promotes intestinal inflammation in mice and counteracts regulatory T cell-mediated protection from colitis. Gastroenterology (2012) 142:335–45. 10.1053/j.gastro.2011.10.027 22057112

[33] Anderson CA Solari R Pease JE Biased agonism at chemokine receptors: obstacles or opportunities for drug discovery? J Leukocyte Biol (2016) 99:901–9. 10.1189/jlb.2MR0815-392R 26701135

[34] Rajagopal S Bassoni DL Campbell JJ Gerard NP Gerard C Wehrman TS Biased agonism as a mechanism for differential signaling by chemokine receptors. J Biol Chem (2013) 288:35039–48. 10.1074/jbc.M113.479113 PMC3853256 24145037

[35] Bennett LD Fox JM Signoret N Mechanisms regulating chemokine receptor activity. Immunology (2011) 134:246–56. 10.1111/j.1365-2567.2011.03485.x PMC3209565 21977995

[36] Dieleman LA Ridwan BU Tennyson GS Beagley KW Bucy RP Elson CO Dextran sulfate sodium-induced colitis occurs in severe combined immunodeficient mice. Gastroenterology (1994) 107:1643–52. 10.1016/0016-5085(94)90803-6 7958674

[37] Boschetti G Kanjarawi R Bardel E Collardeau-Frachon S Duclaux-Loras R Moro-Sibilot L Gut Inflammation in Mice Triggers Proliferation and Function of Mucosal Foxp3+ Regulatory T Cells but Impairs Their Conversion from CD4+ T Cells. J Crohn’s Colitis (2017) 11:105–17. 10.1093/ecco-jcc/jjw125 27364948

[38] Dieleman LA Palmen MJ Akol H Bloemena E Pena AS Meuwissen SG Chronic experimental colitis induced by dextran sulphate sodium (DSS) is characterized by Th1 and Th2 cytokines. Clin Exp Immunol (1998) 114:385–91. 10.1046/j.1365-2249.1998.00728.x PMC1905133 9844047

[39] Kindermann M Knipfer L Atreya I Wirtz S ILC2s in infectious diseases and organ-specific fibrosis. Semin Immunopathol (2018) 40:379–92. 10.1007/s00281-018-0677-x 29623414

[40] Panda SK Colonna M Innate Lymphoid Cells in Mucosal Immunity. Front Immunol (2019) 10:861. 10.3389/fimmu.2019.00861 31134050 PMC6515929

[41] Monticelli LA Osborne LC Noti M Tran SV Zaiss DM Artis D IL-33 promotes an innate immune pathway of intestinal tissue protection dependent on amphiregulin-EGFR interactions. Proc Natl Acad Sci USA (2015) 112:10762–7. 10.1073/pnas.1509070112 PMC4553775 26243875

[42] Gonzalo JA Qiu Y Lora JM Al-Garawi A Villeval JL Boyce JA Coordinated involvement of mast cells and T cells in allergic mucosal inflammation: critical role of the CC chemokine ligand 1:CCR8 axis. J Immunol (2007) 179:1740–50. 10.4049/jimmunol.179.3.1740 17641040

[43] Asai A Nakamura K Kobayashi M Herndon DN Suzuki F CCL1 released from M2b macrophages is essentially required for the maintenance of their properties. J Leukocyte Biol (2012) 92:859–67. 10.1189/jlb.0212107 22730547

[44] Fuchs A Vermi W Lee JS Lonardi S Gilfillan S Newberry RD Intraepithelial type 1 innate lymphoid cells are a unique subset of IL-12- and IL-15-responsive IFN-gamma-producing cells. Immunity (2013) 38:769–81. 10.1016/j.immuni.2013.02.010 PMC3634355 23453631

[45] Penny HA Hodge SH Hepworth MR Orchestration of intestinal homeostasis and tolerance by group 3 innate lymphoid cells. Semin Immunopathol (2018) 40:357–70. 10.1007/s00281-018-0687-8 PMC6060788 29737384

[46] Klose CS Kiss EA Schwierzeck V Ebert K Hoyler T d’Hargues Y A T-bet gradient controls the fate and function of CCR6-RORgammat+ innate lymphoid cells. Nature (2013) 494:261–5. 10.1038/nature11813 23334414

[47] Bank U Deiser K Plaza-Sirvent C Osbelt L Witte A Knop L c-FLIP is crucial for IL-7/IL-15-dependent NKp46(+) ILC development and protection from intestinal inflammation in mice. Nat Commun (2020) 11:1056. 10.1038/s41467-020-14782-3 32103006 PMC7044440

[48] Song C Lee JS Gilfillan S Robinette ML Newberry RD Stappenbeck TS Unique and redundant functions of NKp46+ ILC3s in models of intestinal inflammation. J Exp Med (2015) 212:1869–82. 10.1084/jem.20151403 PMC4612098 26458769

[49] Rankin LC Girard-Madoux MJ Seillet C Mielke LA Kerdiles Y Fenis A Complementarity and redundancy of IL-22-producing innate lymphoid cells. Nat Immunol (2016) 17:179–86. 10.1038/ni.3332 PMC4720992 26595889

[50] Vonarbourg C Mortha A Bui VL Hernandez PP Kiss EA Hoyler T Regulated expression of nuclear receptor RORgammat confers distinct functional fates to NK cell receptor-expressing RORgammat(+) innate lymphocytes. Immunity (2010) 33:736–51. 10.1016/j.immuni.2010.10.017 PMC3042726 21093318

[51] Keir M Yi Y Lu T Ghilardi N The role of IL-22 in intestinal health and disease. J Exp Med (2020) 217:e20192195. 10.1084/jem.20192195 32997932 PMC7062536

[52] Brasseit J Kwong Chung CKC Noti M Zysset D Hoheisel-Dickgreber N Genitsch V Divergent Roles of Interferon-gamma and Innate Lymphoid Cells in Innate and Adaptive Immune Cell-Mediated Intestinal Inflammation. Front Immunol (2018) 9:23. 10.3389/fimmu.2018.00023 29416538 PMC5787534

[53] Nava P Koch S Laukoetter MG Lee WY Kolegraff K Capaldo CT Interferon-gamma regulates intestinal epithelial homeostasis through converging beta-catenin signaling pathways. Immunity (2010) 32:392–402. 10.1016/j.immuni.2010.03.001 20303298 PMC2859189

[54] Gurtner GJ Newberry RD Schloemann SR McDonald KG Stenson WF Inhibition of indoleamine 2,3-dioxygenase augments trinitrobenzene sulfonic acid colitis in mice. Gastroenterology (2003) 125:1762–73. 10.1053/j.gastro.2003.08.031 14724829

[55] Metghalchi S Ponnuswamy P Simon T Haddad Y Laurans L Clement M Indoleamine 2,3-Dioxygenase Fine-Tunes Immune Homeostasis in Atherosclerosis and Colitis through Repression of Interleukin-10 Production. Cell Metab (2015) 22:460–71. 10.1016/j.cmet.2015.07.004 26235422

[56] Nowarski R Jackson R Gagliani N de Zoete MR Palm NW Bailis W Epithelial IL-18 Equilibrium Controls Barrier Function in Colitis. Cell (2015) 163:1444–56. 10.1016/j.cell.2015.10.072 PMC4943028 26638073

[57] Kim CH Hashimoto-Hill S Kim M Migration and Tissue Tropism of Innate Lymphoid Cells. Trends Immunol (2016) 37:68–79. 10.1016/j.it.2015.11.003 26708278 PMC4744800

[58] Willinger T Metabolic Control of Innate Lymphoid Cell Migration. Front Immunol (2019) 10:2010. 10.3389/fimmu.2019.02010 31507605 PMC6713999

