# Supplementary figures and images for: CCR8 Signaling via CCL1 Regulates Responses of Intestinal IFN-γ Producing Innate Lymphoid CelIs and Protects From Experimental Colitis
Source: Front Immunol. 2021 Feb 5;11:609400. doi: 10.3389/fimmu.2020.609400 (PMC7892458; doi:10.3389/fimmu.2020.609400)

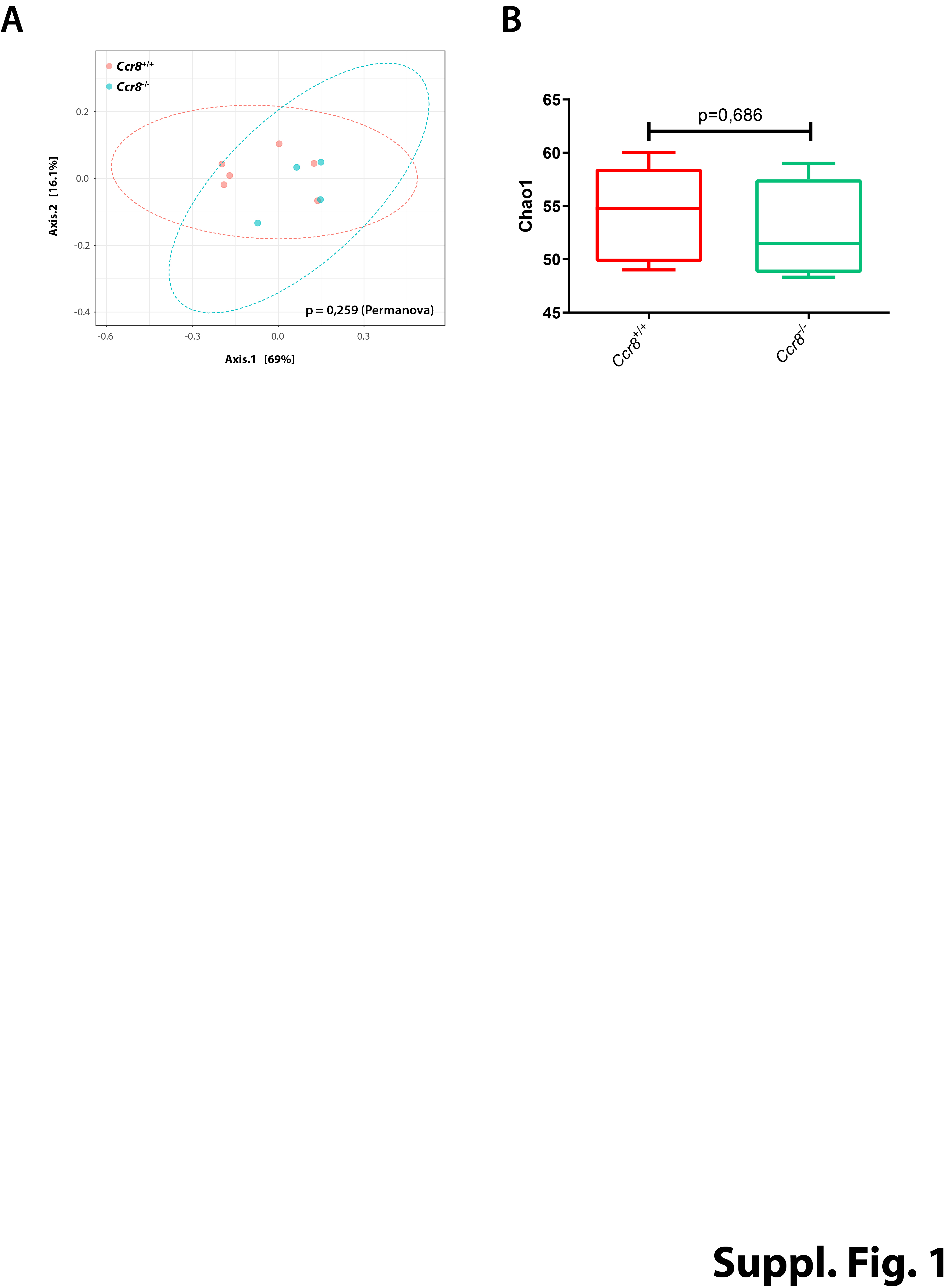

Supplement: Supplementary Figure 1 — Fecal pellets of wildtype and Ccr8−/− mice cohoused for 4 weeks were collected. Genomic DNA was isolated and used for 16S-based metagenomic sequencing. (A) PcoA plot showing beta-diversity (bray curtis dissimilarity). (B) Alpha diversity (Chao1 index). N = 4–6 mice/group. [file Image_1.jpg]

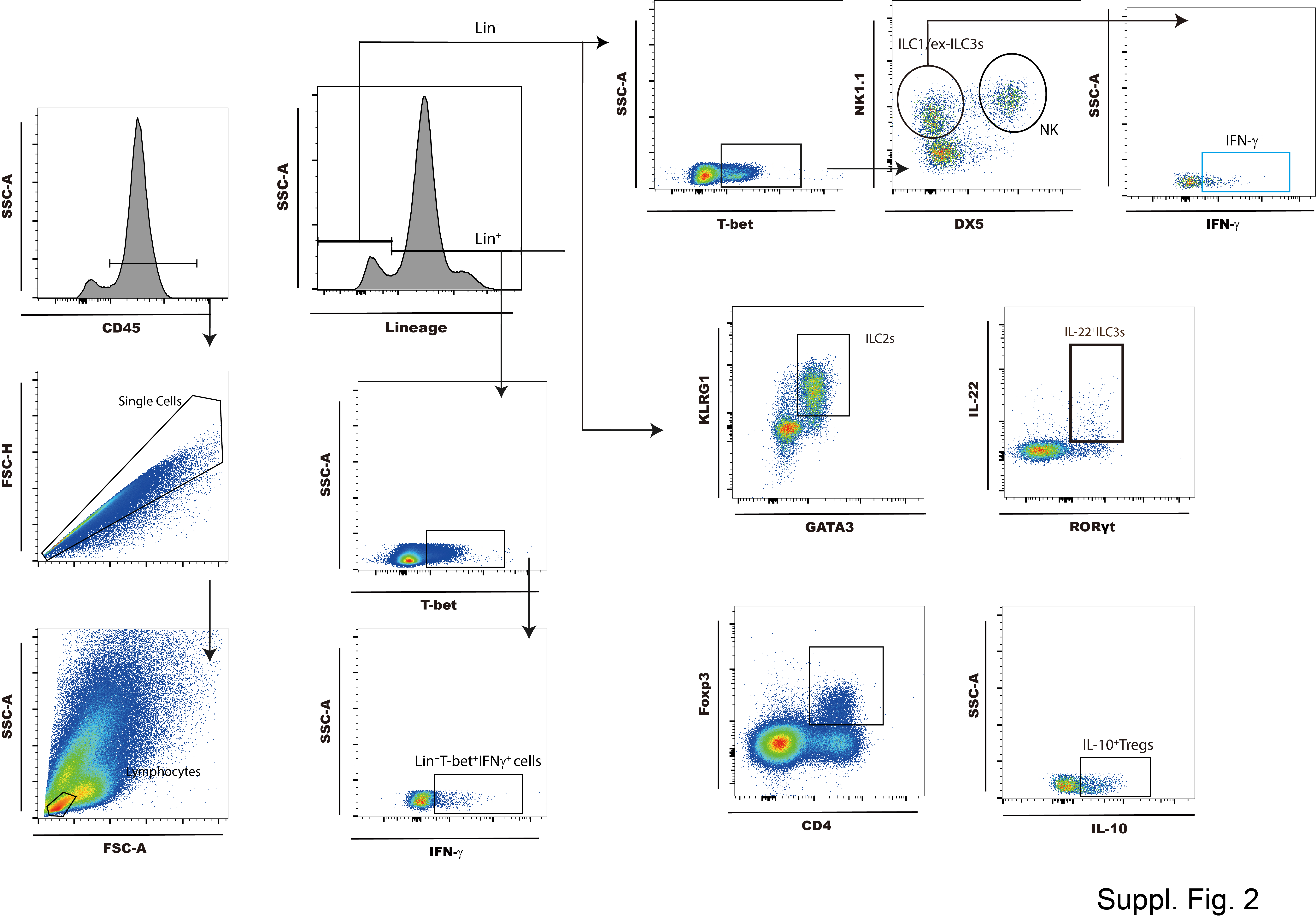

Supplement: Supplementary Figure 2 — Gating strategies for flow cytometric characterization of different lymphocyte populations within the mouse intestinal lamina propria. [file Image_2.jpg]

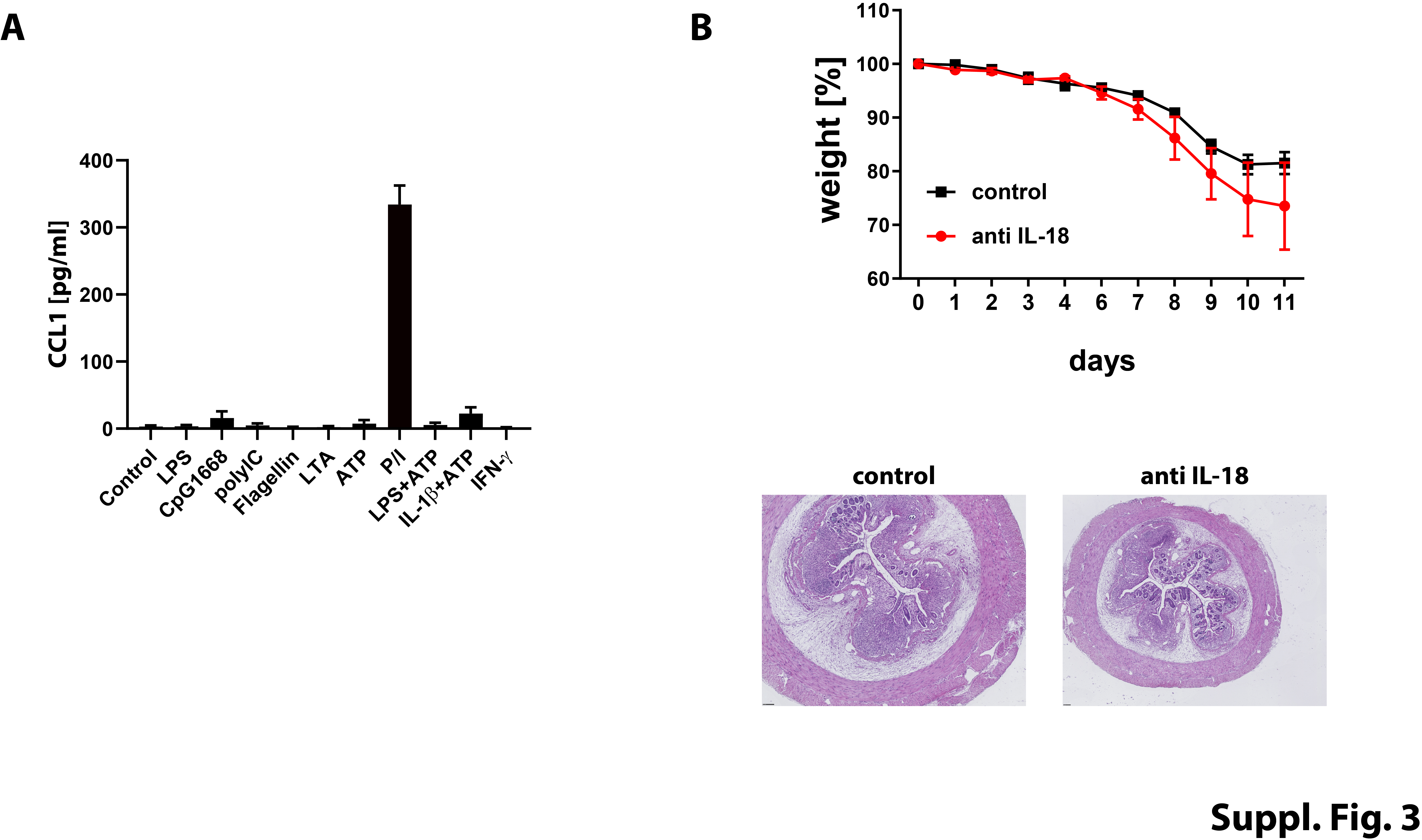

Supplement: Supplementary Figure 3 — (A) The concentrations of CCL1 protein in supernatants of colonic Ccr8 + LPMCs stimulated with LPS (1 µg/ml), CpG1668 (1 µm), polyIC (1 µg/ml), Flagellin (0,1 µg/ml), Lipoteichoic acid (LTA; 2 µg/ml), ATP (2,5 mM), IFN-γ (10 ng/ml) IL-1-β (10 ng/ml) alone or in combination or with Ionomycin and Phorbol 12-myristate 13-acetate (PMA) for 48 h (N = 4–6/group). (B) Ccr8 −/− mice were exposed to 2% of DSS in drinking water for 7 days. At days 4 and 7, they were i.p. treated with 200 mg of anti-IL-18 (clone YIGIF74-1G7) mABs or control antibodies. (N = 4/group). [file Image_3.jpg]
